# Brain Metastases in HER2-Positive Breast Cancer: Current and Novel Treatment Strategies

**DOI:** 10.3390/cancers13122927

**Published:** 2021-06-11

**Authors:** Alejandro Garcia-Alvarez, Andri Papakonstantinou, Mafalda Oliveira

**Affiliations:** 1 Medical Oncology Department, Vall d’Hebron Hospital, 08035 Barcelona, Spain; alejandro.garcia@vhebron.net; 2 Breast Cancer Group, Vall d’Hebron Institute of Oncology (VHIO), 08035 Barcelona, Spain; apapakonstantinou@vhio.net; 3 Department of Oncology-Pathology, Karolinska Institute, 17177 Stockholm, Sweden; 4 Department of Breast Cancer, Endocrine Tumors and Sarcoma, Karolinska University Hospital, 17176 Stockholm, Sweden

**Keywords:** breast cancer, metastatic, HER2, brain metastasis, CNS, trastuzumab, tucatinib, neratinib, lapatinib, pertuzumab

## Abstract

**Simple Summary:**

Development of brain metastases is an important event for patients with breast cancer, and it affects both their survival and their quality of life. Patients with HER2-positive breast cancer are more commonly affected by brain metastases compared to patients with HER2-negative/hormone receptor-positive breast cancer. It is essential to find proper therapies that reduce the risk for metastasis in the brain, as well as agents that are active when metastatic lesions develop. Management of HER2-positive breast cancer has drastically improved in recent years due to the development of several drugs targeting the HER2 receptor. This review aims to provide insight into current and novel treatment strategies for patients with brain metastases from HER2-positive breast cancer.

**Abstract:**

Development of brain metastases can occur in up to 30–50% of patients with breast cancer, representing a significant impact on an individual patient in terms of survival and quality of life. Patients with HER2-positive breast cancer have an increased risk of developing brain metastases; however, screening for brain metastases is not currently recommended due to the lack of robust evidence to support survival benefit. In recent years, several novel anti-HER2 agents have led to significant improvements in the outcomes of HER2-positive metastatic breast cancer. Despite these advances, brain and leptomeningeal metastases from HER2-positive breast cancer remain a significant cause of morbidity and mortality, and their optimal management remains an unmet need. This review presents an update on the current and novel treatment strategies for patients with brain metastases from HER2-positive breast cancer and discusses the open questions in the field.

## 1. Introduction

It is estimated that 30–50% of patients with metastatic breast cancer (MBC) will develop brain metastases (BM) [1,2]. In the Unicancer Epidemiological Strategy and Medical Economics (ESME) MBC database (*n =* 16,701), 24.6% of the patients developed BM and the risk was higher for patients with HER2-positive/hormone receptor (HR)-negative and triple-negative (TNBC) breast cancer [2]. In an individual patient data meta-analysis (*n =* 9524) by the International Breast Cancer Study Group (IBCSG) including patients with early BC with no prior systemic therapy, the 10-year cumulative incidence of BM was higher among women with HER2-positive disease (6.8% versus 3.5%; *p* < 0.01) [3]. Other factors associated with BM were an age older than 70 years, the presence of more than two metastatic sites at MBC diagnosis, HR negativity and a more advanced stage of primary tumor [2,4]. Despite this compelling evidence, screening for BM is currently not recommended due to a lack of data to support its benefit in terms of overall survival [5]. The actual incidence of BM at the time of MBC diagnosis may therefore be higher than reported, given that, in the majority of the cases, BM diagnosis is preceded by neurological symptoms. The potential benefit from proactive screening strategies in selected patients with increased risk for BM is being studied in ongoing clinical trials (NCT03881605, NCT03617341, NCT04030507).

In the contemporary registHER (*n =* 1012) and SystHER (*n =* 997) observational studies that included patients with HER-positive tumors treated with trastuzumab and other anti-HER2 therapies, the incidence of BM was 37.3% [6] and 30.6% [7], respectively. The higher incidence of BM observed in these cohorts could be related to improved radiological detection of BM and longer survival associated with new and more effective HER2-targeted therapies. The aforementioned studies also demonstrated worse overall survival (OS) in patients with HER2- positive MBC with central nervous system (CNS) events compared to those without; 26.3 (*n =* 377) versus 44.6 (*n =* 635) months in the registHER study, and 30 (*n =* 299) versus 38 (*n =* 678) months in the SystHER study [6,7].

The best management of BM in breast cancer is not consensual, but it generally includes a combination of local interventions, such as surgery, whole-brain and/or stereotactic radiotherapy and systemic anticancer therapies [5,8]. In many situations, such as leptomeningeal metastasis, recommendations are practically based on expert opinions [5]. The term blood–brain barrier (BBB) refers to the unique features of the non-fenestrated vessels that vascularize the CNS, which critically interact with mural cells, immune cells, glial cells and neural cells to tightly regulate the movement of ions, molecules and cells between the blood and the brain [9]. While high molecular weight molecules, such as monoclonal antibodies, typically do not cross an intact BBB [10], the latter may be disrupted in patients with BM metastasis and/or with prior CNS-directed therapy [11]. Patients with BM have been largely excluded from enrollment in clinical trials, especially when in progression [12], which has hampered development of novel systemic treatments in this setting.

This review focuses on the activity of the different anti-HER2 agents in patients with BM from HER2-positive MBC and, compared to other recent reviews [13], amounts to an update of the relevant evidence on systemic therapy, alone or in combination with radiotherapy, and it also asks questions about prevention and/or early diagnosis of BM in this specific subgroup of patients. Table 1 summarizes the study characteristics and related outcomes of the articles included in the review.

## 2. Prevention of Brain Metastases

### 2.1. Trastuzumab

Trastuzumab is a monoclonal antibody directed against the extracellular domain (subdomain IV) of HER2 and its discovery has significantly changed the natural history of HER2-positive breast cancer. Despite the transformative impact of trastuzumab in the treatment of patients with early HER2-positive breast cancer, recurrence in the CNS is still a major health issue.

A meta-analysis of randomized trials, including four trials and a total of 9020 patients, showed that adjuvant trastuzumab was associated with an increased incidence of BM as a first site of disease recurrence (1.94% no trastuzumab versus 2.56% trastuzumab, relative risk [RR] 1.35; *p* = 0.038) [14]. In contrast, in the registHER study, treatment with trastuzumab was associated with significant delay of the time to BM (14 versus 7 months) and also improved survival after BM diagnosis (18 versus 4 months) [6]. Similarly, Dawood and colleagues demonstrated a significantly prolonged time to the development of BM in patients with HER2-positive disease who received trastuzumab compared to those who did not (HR 2.13, 95% CI 1.51–3.00; *p* < 0.001) [15]. Taken together, these data suggest that, while extracranial disease is effectively controlled with trastuzumab, an intact BBB may prevent trastuzumab penetration to the CNS. However, it can also be hypothesized that the survival benefit achieved by trastuzumab allows for enough time for patients to develop BM, an event not fully prevented by trastuzumab.

### 2.2. Trastuzumab–Pertuzumab

Pertuzumab is a recombinant monoclonal IgG1 antibody directed against the extracellular dimerization domain (subdomain II) of the HER2 receptor. Adding pertuzumab to trastuzumab and paclitaxel in first-line treatment for patients with unresectable and/or metastatic HER2-positive BC significantly increased progression-free survival (PFS) and OS, as demonstrated by the CLEOPATRA phase III trial [49,50]. Patients with clinical and/or radiographic evidence of BM were excluded from the trial. In an exploratory analysis, it was found that the incidence of BM did not significantly differ between the treatment groups (13.7% in the pertuzumab arm versus 12.6% in the placebo arm). However, pertuzumab significantly increased time to the development of BM (15 versus 11.9 months, respectively, HR 0.58; *p* = 0.0049) and improved survival [16]. The latter can potentially be attributed to the beneficial action of the combination on extracranial disease, intrinsic intracranial activity of pertuzumab or a combination of both.

### 2.3. Ado-Trastuzumab-Emtansine (T-DM1)

T-DM1, an antibody–drug conjugate (ADC) composed of trastuzumab and a derivative of maytansine as cytotoxic payload has been approved by the Food and Drug Administration (FDA) and the European Medicines Agency (EMA) in patients with HER2-positive metastatic disease after progression to trastuzumab and a taxane, and in the adjuvant treatment of patients with HER2-positive early breast cancer who have residual invasive disease after neoadjuvant taxane and trastuzumab-based treatment [21,51,52,53]. In the early setting, the KATHERINE study demonstrated a significant survival benefit provided by adjuvant T-DM1 when administered after failure to achieve pathological complete response (pCR) with neoadjuvant therapy (HR for invasive disease 0.50, 95% CI 0.39–0.64) and the prolongation of time to relapse (invasive disease or death). The incidence of CNS recurrence as a first site of metastasis among patients treated with adjuvant T-DM1 in the study was numerically higher compared to trastuzumab (5.9% versus 4.3%, respectively) [52]. The results underline the efficacy of T-DM1 in this setting and, at the same time, highlight once again the enduring problem with CNS relapse in HER2-positive BC.

In the metastatic setting, T-DM1 significantly improved PFS compared to the lapatinib–capecitabine combination (9.6 versus 6.4 months; HR 0.65, 95% CI 0.55–0.77) [53]. The drug also led to a significantly longer OS, even when considering the 136 patients that had crossed over to T-DM1 (*n =* 136); OS 29.9 versus 25.9 months; HR 0.75, 95% CI 0.64–0.88 [51]. Regarding BM, T-DM1 led to a non-significant reduction of the incidence of BM development, from 2% to 0.7%, when compared to lapatinib plus capecitabine [21].

In summary, while T-DM1 may have the same limitations as trastuzumab in penetrating the BBB (probably due to large molecular weight), there are some encouraging data regarding its intracranial activity. These findings also raise the question as to whether disruption of the BBB could occur prior to the development of radiological evident CNS metastasis in patients with metastatic HER2-positive BC. This could explain the different efficacy of large molecules in primary and secondary prevention of BM.

### 2.4. Lapatinib

Lapatinib is a reversible dual tyrosine kinase inhibitor (TKI) targeting the epidermal growth factor (EGFR, ErbB-1) and HER2 (ErbB-2), and was initially approved for combination with capecitabine in HER2-positive MBC [54,55]. Several other studies have since investigated lapatinib in the neoadjuvant and metastatic settings in various combinations with other HER2-inhibitors. Given the theoretically higher CNS penetration of a TKI compared to an antibody, lapatinib activity has been explored in patients with CNS disease, with two main objectives: prevent CNS relapse (CEREBEL) [28] and explore its efficacy in patients with CNS metastasis (EGF105084, LANDSCAPE) [29,31].

CEREBEL was a pivotal randomized phase III trial that compared lapatinib and capecitabine with trastuzumab and capecitabine in patients with HER2-positive MBC with no BM at enrollment [28]. The primary endpoint of the trial was incidence of CNS as site of first relapse, based on independent review committee assessment of brain MRI scans. The study was terminated prematurely after the inclusion of 540 patients and demonstrated no significant difference between the groups in terms of incidence of BM (odds ratio [OR] 0.65, 95% CI 0.26–1.63), time to first relapse in the brain (5.7 versus 4.4 months) or progression in the brain at any time (OR 1.14, 95% CI 0.52–2.51) [28]. In the overall population, trastuzumab plus capecitabine demonstrated improved PFS and OS. Although the number and type of prior anti-HER2 regimens may have impacted lapatinib performance, the trial was probably underpowered to detect an existing difference due to its premature termination.

### 2.5. Novel Agents

#### Neratinib

Neratinib is an irreversible pan-HER TKI and has been approved for the management of both early and metastatic BC. The NEfERT-T phase III trial compared neratinib plus paclitaxel versus trastuzumab plus paclitaxel. Patients with BM were eligible if the CNS lesions were asymptomatic [36]. Median PFS in both treatment groups was 12.9 months (HR 1.02, 95% CI 0.81–1.27) but the neratinib–paclitaxel combination led to an increased incidence of grade 3 diarrhea (30.4%) compared to trastuzumab–paclitaxel (3.8%). Despite increased toxicity and lack of PFS benefit, treatment with neratinib plus paclitaxel halved the incidence of CNS recurrence compared to trastuzumab plus paclitaxel (relative risk 0.48, 95% CI 0.29–0.79), and also prolonged the time to development of BM (HR 0.45, 95% CI 0.26–0.78) [36]. Despite fewer patients with BM at baseline in the neratinib–paclitaxel group, the benefit from neratinib remained after adjusting for this difference, regardless of baseline BM status [36]. For the interpretation of these results, it is worth noting that, as screening for synchronous BM at baseline was not performed, the number of metachronous CNS events may have been underestimated.

## 3. Systemic Therapy in the Treatment of Brain Metastases

### 3.1. Trastuzumab

As mentioned earlier, trastuzumab has been generally considered not to cross the BBB due to its high molecular weight. However, in animal models with HER2-positive BM, trastuzumab uptake in the brain and reduction in the BM has been observed [56,57]. Physical disruption of the BBB due to BM growth and increased blood vessel permeability derived from neovessel formation and vascular endothelial growth factor (VEGF) secretion may explain the antibody’s brain penetration [58]. However, these observations have not been translated into significant efficacy of single agent trastuzumab in BM in vivo [59,60].

Aiming to improve permeability to the CNS, new mechanisms to disrupt the BBB are being investigated. An example is NEO100, a highly pure derivate of the natural monoterpene perillyl alcohol [61] that has been shown to increase trastuzumab and T-DM1 capacity to penetrate BBB in vitro and in vivo [62]. Intraarterial administration of NEO100 in murine models also facilitated the entry of both drugs into the brain. This was translated into significantly higher apoptotic cell death activity and longer survival for the mice that were treated with the NEO100 combination compared to either anti-HER2 drugs alone or no treatment [62].

Despite a lack of compelling evidence to support trastuzumab monotherapy, some data imply some degree of activity, even for this agent. The already-mentioned registHER trial demonstrated prolonged survival (17.5 versus 3.7 months, HR 0.25, 95% CI 0.20–0.33, *p* < 0.001) among patients with BM treated with trastuzumab (*n =* 258), compared to no trastuzumab (*n =* 119) [6]. A multivariate analysis also identified trastuzumab as an independent favorable prognostic factor for the risk of death (HR 0.33, 95% CI 0.25—0.46, *p* < 0.001) [6]. Interestingly, radiotherapy was not independently related to improved survival in this cohort. Park et al. also reported survival benefit with trastuzumab in 77 patients with HER2-positive BC with BM [63]. In this retrospective cohort, administration of trastuzumab (*n =* 42) led to prolonged time to death from BM (14. 9 versus 4 months, *p* = 0.0005) [63]. Nevertheless, the superiority of a combination of anti-HER2 therapies, rather than a trastuzumab monotherapy, even in extracranial disease, is undisputable and is currently the strategy of choice.

### 3.2. Trastuzumab-Pertuzumab

The phase II PATRICIA trial investigated the efficacy of pertuzumab and high-dose trastuzumab (6 mg/kg weekly) in patients who had experienced progression of BM after CNS radiotherapy but maintained stable extracranial disease. Among the 39 analyzed patients, 11% (95% CI 3–25%) experienced intracranial objective response according to the Response Assessment in Neuro-Oncology Brain Metastases working group (RANO-BM), with a median duration of response (DOR) of 4.6 months [17]. The clinical benefit rate (partial response + complete response + stable disease at 6 months) was 51% [18]. Notably, exploratory studies demonstrated a higher concentration of trastuzumab in serum with this treatment schedule compared to historical controls, suggesting a dose-dependent relationship. Given the limited toxicity reported by the authors, this study provides the possibility for another treatment approach, although more investigations are required to better describe the biological background of the dose-dependency, as well as to validate the toxicity and efficacy results.

Activity on BM was also reported in the RePer study, a retrospective observational study of 264 patients with HER2-positive MBC aiming to explore the efficacy of first-line treatment with pertuzumab/trastuzumab/taxane in a real-world setting. Dual HER2-blockade and taxanes resulted in objective response rate (ORR) in 52% of the patients with BM at baseline (*n =* 21) compared to 82% in those without BM (*n =* 98). In the whole cohort, 13% of the patients without previous BM experienced progress in the CNS (*n =* 33) and had worse PFS and two-year OS compared to the patients with BM at base-line; 20 versus 13 months and 77.7% versus 65.6%, respectively [18]. In a similar study, median PFS and OS among 13 patients with BM at baseline treated with first-line trastuzumab/pertuzumab and taxane was 16.8 months and 26.7 months, respectively [19]. Of note, all patients were trastuzumab naïve; thus, this study provides information on the activity on BM of upfront treatment with pertuzumab and trastuzumab.

Benefits from combining the two antibodies with other chemotherapy regimens have also been reported. In the PHEREXA phase III trial, the addition of pertuzumab to capecitabine and trastuzumab significantly improved PFS in the subgroup of patients with BM at baseline (*n =* 53; HR 0.29, 95% CI 0.15–0.60) [20]. Similarly, although not designed to answer the question of CNS progression, the EORTC 75111-10114 trial enrolled 80 older patients and showed numerical differences in brain-only relapse between treatment with pertuzumab and trastuzumab alone (*n =* 2; 5%), as well as a combination with metronomic oral cyclophosphamide (*n =* 4; 10%) [64].

In summary, these early studies indicate some activity of the trastuzumab–pertuzumab doublet in BM, always in combination with other agents, and warrant further evaluation.

### 3.3. Ado-Trastuzumab-Emtansine (T-DM1)

The role of T-DM1 in intracranial disease has been assessed in a post hoc analysis of the phase IIIb KAMILLA study [22]. The study enrolled 398 patients with baseline BM, 56.8% of whom had had prior radiotherapy. Among the 126 patients with measurable disease, ORR was 21.4% (95% CI 14.6–29.6) and median PFS and OS were 5.5 (95% CI 5.3–5.6) and 18.9 (95% CI 17.1–21.3) months, respectively. Compared to patients with no BM at baseline, OS was significantly worse among patients with known BM at study enrollment, even after adjustment for other risk factors (adjusted HR 1.18, 95% CI 1.02–1.38) [22]. Interestingly, 67 patients with new brain lesions continued TDM-1 post-progression and had a clinically relevant duration of T-DM1; 8.8 months (0–37 months) and 6.2 months (1–28 months) among patients with and without baseline BM, respectively [22]. Furthermore, in the EMILIA trial (T-DM1 versus lapatinib-capecitabine), no difference in CNS progression rate (22.2% and 16%) and median PFS (5.9 versus 5.7 months) was observed among the 95 patients with previously treated and stable CNS metastases at baseline [21].

In addition, in a smaller observational study (*n =* 10), objective response was observed in three patients, and median PFS in the CNS was 5 months (95% CI 3.69–6.32 months) [23]. In another study including patients with BM treated with T-DM1 (*n =* 53), ORR was 25%, PFS was 7 months (95% CI 5.4–8.6 months) and OS was 14 months (95% CI 12.2–15.8) [24]. Finally, another retrospective series (*n =* 39) also demonstrated clinical benefit in over 50% of the patients and a PFS of 6.1 months (95% CI 5.2–18.3 months), which is in line with the PFS data reported in the KAMILLA study [25].

To summarize, T-DM1 appears to have some activity in the context of BM, with PFS around 5–7 months, although OS remains significantly inferior compared to patients without intracranial disease.

### 3.4. Lapatinib

Lapatinib as monotherapy in patients with progressing BM and prior trastuzumab and radiotherapy was tested in the EGF105084 trial with discouraging results [29]. Only 6% among 242 patients experienced a partial response, and PFS (2.4 months, 1.87–2.79) and OS (6.37, 5.49–8.25) were poor [29]. Interestingly, 20% (*n =* 10) of the 50 patients that switched to lapatinib plus capecitabine after progression on lapatinib monotherapy achieved a partial response. Likewise, as shown by Blackwell and colleagues, combining lapatinib with trastuzumab led to numerically less progressions of BM compared with lapatinib monotherapy (9/16 and 15/20, respectively), but the numbers are too small for a formal comparison [30]. Similarly, the combination was superior to both lapatinib and trastuzumab monotherapy in a retrospective cohort by Hayashi et al. [65].

As shown above, the lapatinib–capecitabine doublet did not significantly prevent development of BM, but the combination could benefit patients with established, previously untreated BM, as reported in the LANDSCAPE trial [31]. Among 45 enrolled patients, 29 experienced a partial response (65.9%, 95% CI 50.1–79.5%) and median time to progression was 5.5 months, with a significantly longer time to progression among responders (6 months, 95% CI 5.5–7.4) than non-responders (2.8, 95% CI 1.4–4.2). Moreover, in other small cohorts, the doublet yielded a volumetric ORR of 20–38% in patients progressing after prior radiotherapy [32,33]. A pooled analysis performed by Petrelli et al. also demonstrated objective response in about one third of the patients with BM treated with the combination of lapatinib and capecitabine (ORR 29.2%, 95% CI 18.5–42.7) [66]. The ORR was, however, only 18.7% when studies exclusively including patients with progressive BM were analyzed, showing the importance of timely diagnosis and management of BM [66].

In brief, despite the theoretical increased penetrance of a TKI in the CNS, monotherapy with lapatinib has not demonstrated significant intracranial activity. Lapatinib also seems to be more active when given in combination with other agents, such as capecitabine, especially in the context of untreated BM.

### 3.5. Novel Agents

#### 3.5.1. Trastuzumab Deruxtecan

Trastuzumab deruxtecan (also known as fam-trastuzumab deruxtecan or DS-8201a) is an ADC composed of a humanized monoclonal antibody anti-human HER2 attached to a topoisomerase I inhibitor payload by a tetrapeptide-based, enzyme-cleavable linker. Compared to T-DM1, trastuzumab deruxtecan has a higher antibody-drug ratio (8 versus 3–4) and is probably more potent than T-DM1 as a result of the properties of its payload that facilitate penetration of the cell membrane of the target or neighboring cells, without requiring high receptor expression [67]. Efficacy of DS-8201a has been assessed in the DESTINY-Breast01 phase II trial that included 184 patients with unresectable or metastatic HER2-positive breast cancer previously treated with T-DM1 [26]. Among this heavily pre-treated population of patients, trastuzumab deruxtecan achieved an ORR of 60.9% and a median PFS of 16.4 months (95% CI 12.7 to not reached) [26].

Patients with BM were eligible for inclusion in the DESTINY Breast01 trial if the brain lesions were previously treated and/or controlled. Among 24 patients with baseline BM, ORR was 58.3% (95% CI 36.6–77.9) and median PFS was 18.1 months (95% CI 6.7–18.1). Progression in the brain was noted in 8% of the patients with baseline BM (*n =* 2/24) and in 1.3% of the patients without BM at baseline (*n =* 2/160) [26,27].

Two ongoing studies, NCT04752059 (TUXEDO-1) and NCT04739761 (DESTINY B12), are prospectively enrolling patients to assess the efficacy of trastuzumab deruxtecan in patients with BM. The former is a small phase II study and is already recruiting patients, whereas the latter is a single-arm phase IIIb/4 trial that will include two separate cohorts, distinguishing patients with and without BM, but has not get begun patient enrollment. Both trials will include patients with HER2-positive BC with newly diagnosed or progressing BM and measurable disease according to the RANO-BM criteria. The TUXEDO-1 trial allows for previous treatment with T-DM1, whereas the DESTINY B-12 trial excludes patients with prior treatment with tucatinib. The results of these studies are expected to shed more light on the intracranial activity of trastuzumab deruxtecan.

#### 3.5.2. Neratinib

In the metastatic setting, neratinib was investigated in the phase II TBCRC022 trial in three cohorts: neratinib as a single agent, neratinib as a single agent in patients undergoing surgical excision of CNS lesions and neratinib in combination with capecitabine [34]. In the first cohort, 40 patients were enrolled, 31 of whom had received prior WBRT. The ORR in the CNS was 8% (95% CI, 2% to 22%) and the median PFS was 1.9 months. The third cohort (neratinib plus capecitabine) enrolled 49 patients with HER2-positive BM that had progressed after local treatment in the CNS. Half of the patients in the lapatinib-naïve group (composite CNS ORR 49%, 95% CI 32–66%) and one third among those previously exposed to lapatinib experienced an objective response (composite CNS ORR 33%, 95% CI 10–65%). Median PFS in cohort 3A and 3B was 5.5 months (range 0.8–18.8) and 3.1 months (range 0.7–14.6), respectively, and median OS was 13.3 (range 2.2–27.6) and 15.1 months (range 0.8–23.7), respectively [34]. In spite of some imbalance in the number of previous therapy lines, OS was similar, regardless of prior lapatinib treatment.

Neratinib and capecitabine were compared to lapatinib–capecitabine in the phase III NALA trial. The neratinib combination was not only superior in terms of PFS, but also demonstrated intracranial activity with significantly lower incidence of BM; 22% (95% CI 15.5–30.9%) versus 29.2% in lapatinib–capecitabine (95% CI 22.5–36.1%; HR 0.6, 95% CI 0.6–1.01) [35]. Interestingly, fewer patients in the neratinib arm required intervention for symptomatic CNS metastases (22.8% versus 29.2%, *p* = 0.043), suggesting an effect of neratinib in the delay of CNS progression, or at least relief of symptoms [35]. Based on these results, neratinib–capecitabine is a preferred combination over lapatinib–capecitabine, although drug availability and reimbursement issues may be an issue.

#### 3.5.3. Tucatinib

Tucatinib is an oral tyrosine kinase inhibitor that is highly selective for the kinase domain of HER2, with minimal inhibition of epidermal growth factor receptor.

The pivotal HER2CLIMB phase III trial enrolled patients with HER2-positive metastatic breast cancer previously treated with trastuzumab, pertuzumab and T-DM1, and randomized to receive trastuzumab, capecitabine and tucatinib or placebo [68]. The addition of tucatinib to trastuzumab and capecitabine led to improved PFS (7.8 versus 5.6 months; HR 0.54, 95% CI 0.42–0.71; *p* < 0.001), ORR (40.61% versus 22.83%; *p* < 0.001) and OS (21.9 versus 17.4 months; HR 0.66, 95% CI 0.50–0.88; *p* = 0.005). Importantly, the HER2CLIMB trial enrolled 291 patients with BM that were either treated and stable or active (newly diagnosed or progressing after prior local therapy). In patients with BM, PFS at 1 year was 24.9% in the tucatinib group versus 0% in the placebo group (HR 0.48, 95% CI, 0.34–0.69; *p* < 0.001), and the median PFS was 7.6 months (95% CI 6.2–9.5) and 5.4 months (95% CI 4.1–5.7), respectively. Tucatinib also demonstrated clinically meaningful activity with prolonged CNS-specific PFS (9.9 months versus 4.2 months in the control group; HR 0.32, 95% CI 0.22–0.48, *p* < 0.0001), OS (18.1 versus 12.0 months; HR 0.58, 95% CI 0.40–0.85, *p* = 0.005) and intracranial ORR (47.3% versus 20.0%; *p* = 0.03) [37]. As per protocol, patients with isolated progression in the brain could continue study therapy after local treatment. Among 30 patients who continued post-progression, the median time from randomization to second disease progression or death was 15.9 months with tucatinib versus 9.7 months in the control group (HR 0.33, 95% CI 0.11–0.02).

Tucatinib has also shown signs of activity in BM in combination with trastuzumab or T-DM1 in phase I studies [38,39]. In a phase I dose-escalation trial of tucatinib with a maximum tolerated dose of 300 mg twice a day (cohort A) or 750 mg once daily (cohort B) in combination with trastuzumab in patients with BM, seven patients in both cohorts together had a clinical benefit rate at 24 weeks [38]. Two of 17 patients in cohort A and one of 17 patients in cohort B experienced partial response, whereas 10 and 16 patients, respectively had stable disease. In another phase I study of tucatinib in combination with T-DM1, 30 of 50 patients treated with 300 mg of tucatinib twice daily had BM at baseline [39]. Median PFS for these patients was 6.7 months (95% CI 4.1–10.2 months) and brain-specific ORR among the 14 patients with measurable BM was 36% [39]. The most effective and/or convenient dose schedule has not been established yet, but the combinations warrant further investigation in, for example, the ongoing NCT04512261 trial.

Tucatinib is, thus far, the first TKI to demonstrate improved antitumor activity against BMs in patients with HER2-positive breast cancer in a randomized controlled trial, and it will probably become an integral part of the management of these patients.

#### 3.5.4. Pyrotinib

Pyrotinib is an oral irreversible inhibitor of the tyrosine kinase activity of EGFR (ErbB-1), HER2 (ErbB-2) and HER4 (ErbB4) [69]. The combination of pyrotinib and capecitabine in the PHENIX phase III trial significantly increased PFS from 4.2 months with single agent capecitabine to 6.9 months in the combination group among the 31 study participants with BM [40].

In a subgroup of 31 patients with BM from real world data reporting on pyrotinib monotherapy or in combinations (*n =* 122), PFS was 6.7 months (both intra- and extracranial lesions considered) and ORR was 28% [41]. Nevertheless, concomitant local treatment (radiotherapy or surgery) improved response, as seen by the difference in ORR in patients with or without local interventions; 66.6% (*n =* 9) and 6.3% (*n =* 16), respectively. Of note, three patients in the first subgroup (33.3%) treated with pyrotinib and capecitabine and concomitant radiotherapy achieved a complete response [41].

Early results from pyrotinib studies are encouraging, and it remains to be seen whether pyrotinib will be added to the armory of anti-HER2 agents.

#### 3.5.5. Margetuximab

Margetuximab is a chimeric IgG1 monoclonal antibody that holds the same epitope specificity of trastuzumab. However, its fragment crystallizable (Fc) domain has been modified to improve affinity for CD16A and reduce affinity for CD32B, resulting in better ADCC activity over trastuzumab in vitro [70].

The antibody has been investigated in the phase III SOPHIA trial that compared margetuximab to trastuzumab, both in combination with chemotherapy (investigator’s choice: capecitabine, eribulin, gemcitabine, or vinorelbine) [71]. Patients were eligible if they had progressed after at least two prior HER2-targeted therapies. Patients with BM were allowed if the metastases were treated and stable. A total of 536 patients were enrolled, and 266 (including 37 patients with BM) and 270 patients (including 34 patients with BM) were randomized to margetuximab and trastuzumab combinations, respectively. The margetuximab group demonstrated significantly improved PFS from 4.9 to 5.8 months (HR 0.76, 95% CI 0.59–0.98; *p* = 0.03). However, the clinical relevance of this improvement of less than a month is debatable. No statistically significant difference was observed in terms of OS (21.6 versus 19.8 months in the margetuximab and trastuzumab groups, respectively), although the final OS analysis is pending [71].

No BM specific endpoints have been reported so far; therefore, it remains unknown whether margetuximab is effective in BM for HER2-positive breast cancer.

### 3.6. Combination of Systemic Therapy with Radiotherapy

Although the anti-HER2 tyrosine kinase inhibitors (TKIs) appear to offer better intracranial disease control compared to the antibodies, local interventions will probably continue to be required for local control and symptom relief. Data informing on the efficacy and safety of anti-HER2 therapies plus radiotherapy (whole brain radiotherapy [WBRT], stereotactic external beam radiotherapy [SBRT] or stereotactic radiosurgery [SRS]) are scarce.

#### 3.6.1. Trastuzumab

Trastuzumab’s combination with WBRT in a small cohort of 31 patients appeared to be safe and effective, with an intracranial ORR of 74.2% and a median time to BM progression of 10 months [42]. No comparative results to either trastuzumab or WBRT alone are available; thus, it remains unclear whether the combination is indeed more beneficial than any of the interventions alone.

#### 3.6.2. T-DM1

Among 12 patients receiving T-DM1 either sequentially to SRS (*n =* 8) or concomitantly with WBRT (*n =* 4) at Institut Curie, ORR was 50% in both groups and median PFS was 5.5 and 12.5 months, respectively. However, radiation necrosis was a common adverse event and it was observed in 30.8% of the patients [43]. This finding is in concordance with an independent cohort of 23 patients treated with T-DM1 in combination with SRS concurrently (*n =* 16) or sequentially (*n =* 7) [44]. Radiation necrosis was then observed in 39.1% of patients compared with 2.5% of 22 patients who did not receive T-DM1. Hence, T-DM1 significantly increased the risk of radiation necrosis by 13.5 times, with a median time from radiotherapy to symptom debut of 16 months (range 1–79) [44]. Preclinical data suggest that DM1 bystander effect over reactive astrocytes enhances Aquaporin 4 expression in previously irradiated human astrocyte cell cultures. Furthermore, T-DM1 also increased radiation-related astrocytic cell death. These results were not seen with trastuzumab, suggesting this adverse event is associated with T-DM1 [44].

#### 3.6.3. Lapatinib

The safety and efficacy of combining anti-HER2 therapies with radiotherapy have been addressed in a phase I trial with lapatinib [45]. A total of 35 women with HER-positive MBC with BM (not exclusively) were enrolled, 28 of them with intracranial measurable disease. The combination demonstrated ORR of 79% (59–92%), much higher than the limited response rate that was observed in lapatinib monotherapy [29,45]. Median PFS was also longer when lapatinib was combined with radiotherapy; 4.8 months compared to the previously mentioned 2.4 months in lapatinib monotherapy [29,45]. However, toxicity was a major limiting factor in this cohort [45], even though main toxicities, such as diarrhea, acneiform rash and asthenia, were attributed to lapatinib. No additional toxicity emanating from the combination was observed.

Local disease control with the combination of lapatinib and SRS was investigated retrospectively in a cohort of 126 patients with BM, 47 of whom received concurrent or sequential lapatinib [46]. The combination significantly decreased local failure and increased OS, with greater benefit in local control among patients with smaller lesions. However, data regarding intracranial response rate were not reported. Concurrent or sequential lapatinib was found to be safe and did not significantly increase cumulative incidence of radiation necrosis [46]. In another retrospective cohort study from the same center, lapatinib in combination with SRS (*n =* 18) significantly increased intracranial complete response rate (35% versus 11%), without significant benefit in terms of ORR (75% versus 57%) or brain target lesion-progression rate (25% versus 43%) compared to SRS alone (*n =* 66) [47]. Furthermore, in a phase II study of lapatinib in combination with WBRT reported by Christodoulou et al. (*n =* 21 breast cancer patients), HER2-positive status (*n =* 12) was related to a longer time to progression (HR 0.18, 95% CI 0.06–0.54) [48].

In light of recent studies showing beneficial activity by anti-HER2 drug combinations containing tucatinib or neratinib, and a lack of comparative data, it is impossible to assess the proper timing and place, if any, of the lapatinib/radiotherapy combination, or to state whether the novel TKIs would be more efficient in this context.

## 4. Challenges and Open Questions

The available data indicate remarkable intracranial efficacy from novel agents, such as tucatinib, trastuzumab deruxtecan and neratinib, even though lapatinib-containing combinations may still be relevant in this context. Treatment-induced diarrhea may be a limiting factor and requires proper surveillance and support. However, despite the major steps of the last decade and the cascade of new anti-HER2 agents that are being developed, some of them with remarkable intracranial efficacy, some questions still remain unanswered.

The guidelines for the management of advanced breast cancer do not support BM screening in patients with HER2-positive breast cancer. Nonetheless, as described in the current review, patients with de novo BM have different characteristics and better outcomes compared to those diagnosed with BM during the course of treatment [72]. In addition, there are, today, more treatment choices in HER2-positive BC with intracranial lesions than before, predisposing a higher probability of clinical benefit from early interventions. Hence, early detection may be crucial for patient survival and welfare. Hopefully, the ongoing NCT03881605, NCT03617341 and NCT04030507 trials will shed some light on this critical question.

On the other hand, the management of early HER2-positive BC has changed drastically during recent years, and there are currently no data to inform of the risk of BM among patients with pCR after neoadjuvant therapy or without pCR treated with adjuvant T-DM1. Similarly, it is unknown whether BM as a first site of distant recurrence presented after neoadjuvant double HER2-blockade, with or without subsequent adjuvant T-DM1, or after adjuvant dual blockade, will demonstrate the same response to the anti-HER2 therapies as the ones demonstrated thus far. All patients included in the DESTINY Breast01 trial had received prior trastuzumab and T-DM1, and about one third of the patients in the NALA study were previously treated with trastuzumab, pertuzumab and T-DM1 [26,35]. Thus, there is compelling evidence to suggest that these new agents have intracranial activity among pretreated patients, but the best timing for the administration of each agent and the optimal sequence remain uncertain.

In addition to elaborating on the preferred and more beneficial sequence of the various anti-HER2 agents, there is a great need for adjuvant studies investigating the role of the novel TKIs in preventing brain metastases. Both reduction in the incidence of BM and/or prolongation of time to development of BM would be of interest in this patient group.

In general, patients with BM have been excluded from clinical trials due to poor performance status, shorter expected survival and fears of increased toxicity [73]. Furthermore, investigators have been reluctant to include these patients due to technical obstacles such as limitations in the application of common assessment criteria such as RECIST or the need for additional imaging with different modalities. Additionally, matters related to patient history, such as concomitant corticosteroids and previous brain radiotherapy, may impede proper assessment in the context of a clinical trial [74]. Even more difficult is the evaluation of patients with a leptomeningeal disease, since the nature of the condition and the variety of symptoms hamper objective radiological size measures and reproducibility [75]. As evidence informing the intracranial activity of the new TKIs and ADC grows, clinical trials no longer exclude patients with BM by default, although enrollment of patients with progressive BM is still uncommon [76]. In the same spirit, the FDA issued a guidance for the industry last year, encouraging the inclusion of patients with BM, with some reservations for investigational drugs expected to have CNS toxicity (Guidance document, cancer clinical trial eligibility criteria: brain metastases, July 2020. Docket number: FDA-2019-D-0357, draft published 13 March 2019).

To extract information that could change practice in this area, it is not enough to only include patients with BM in the studies. Many trials do not have pre-specified efficacy outcomes or independent cohorts for this important subgroup, nor do they make a distinction between dissemination in the brain metastases and meninges. Although some trials assess intracranial activity, BM-related outcomes are usually not included as primary or co-primary endpoints. A summary of ongoing trials with endpoints related to BM is presented in Table 2. This notwithstanding, BM response criteria are different across various clinical trials, hampering comparison and conclusions. Standardization of procedures, endpoints and assessment tools is mandatory in order to facilitate critical appraisal of the studies and inference on the reported outcomes. With that aim, in 2015, the RANO-BM working group established recommendations for response evaluation in clinical trials [74]. The use of a common language should aid determination and discovery of clinical, biological and/or radiomic biomarkers predictive of CNS response, and enable tailoring of systemic and/or local treatments.

The heterogeneity of the clinical presentation of metastatic CNS disease also needs to be addressed. For example, leptomeningeal disease could also be present with or without parenchymal metastases in patients with BC; similarly, progression can refer to new lesions in the brain or in the meninges. Leptomeningeal disease can present with or without cancer cells in the cerebrospinal fluid, and is more uncommon than brain metastases in HER2-positive MBC [77,78]. Endpoints reporting on CNS disease outcomes do not usually distinguish between parenchymal metastases and leptomeningeal disease, thus providing insufficient data about the efficacy of anti-HER2 treatment in patients with leptomeningeal disease, either isolated or in combination with BM [5,77].

Symptoms related to BM can be invalidating for the individual patient, which has special relevance in the context of improved survival in patients with HER2-positive BC. This dictates the need to address both disease-related symptoms and adverse events related to the treatment. It is important to report such aspects in a standardized manner in order to facilitate comparisons between the various agents and their impact on the global health of the patient [5]. For example, administration of neratinib in the TBCRC022 trial was accompanied with deterioration in quality of life, whereas no such relationship was observed in the NALA trial [34,35]. In this context, the use of the same standardized and validated questionnaire by both studies (EORTC QLQ-C30) allows for a more comprehensive comparison. TBCRC022 also demonstrated a decline in neurocognitive function of patients, another key parameter to be evaluated in the specific cohort [34]. Thus, inclusion of patient-reported outcomes, evaluation of neurocognitive function and quality of life aspects as endpoints in future trials are essential.

Taking into consideration the particularities of the CNS, alternative administration routes definitely merit formal investigation. Intrathecal trastuzumab is thought to have antitumoral activity in patients with leptomeningeal disease. A pooled analysis reported a response rate of 66.7%, with a median CNS-PFS of 7.5 months [79]. In another meta-analysis, clinical improvement was reported in 55% and stabilization in 14%, whereas median CNS-PFS was 5.2 months and median OS 13.2 was months [79]. Whether intrathecal administration of trastuzumab–pertuzumab or administration of ADC would provide better responses is far from explored, as is whether this administration route, with the inconvenience and the risks it involves, is more beneficial than the commonly used intravenous and oral routes.

Providing solid guidelines in this complex situation is difficult considering the heterogeneity of assessed outcomes, the backgrounds of included patients, the limited number of comparative studies and the disparities between countries in terms of drug availability and reimbursement of the novel drugs. The European Society for Medical Oncology (ESMO) guidelines, published last year, suggest considering the combination of tucatinib/trastuzumab/capecitabine in patients with HER2-positive BC with BM [5]. Although tucatinib is now approved by the FDA and the EMA, the drug is not globally available. Other combinations such as neratinib/capecitabine or lapatinib/capecitabine can also be of value in this setting, and results from novel drugs, such as trastuzumab deruxtecan, have thus far been promising and could provide more treatment options. The ideal sequence for using all the available agents is unclear at this time, but it is generally accepted that exposure to the different available anti-HER2 agents may improve patients’ outcomes and should be pursued whenever possible.

## 5. Conclusions

BM is a major cause of morbidity and mortality in patients with MBC [1]. To date, local management, either ablative (surgery) or palliative (radiotherapy), has been the preferred option in the management of BM. On the other hand, these treatments are not exempt from side effects, which may further deteriorate new neurological function [60]. Aside from anti-HER2 monoclonal antibodies, TKIs and ADCs have exhibited antineoplastic intracranial activity, either as a response to established CNS metastases or by delaying time in the development of BM. These treatments may substitute, delay or complement local treatment strategies in order to attain the best control of CNS disease. Novel anti-HER2 therapies, clinical trials with CNS disease-specific cohorts, standardization of endpoints and evaluation criteria (both in prevention and in established BM), as well as screening for BM at baseline, are fundamental elements in order to improve management, symptom control and prognosis of HER2-positive MBC patients.

## Figures and Tables

**Table 1:** Characteristics of studies reporting on outcomes related to brain metastases in patients with HER2-positive breast cancer.

Anti-HER2 Agent	Study	Type of Study	Population	Treatment	Reported Outcomes Related to BM
Trastuzumab					
	RegistHER, Brufsky et al., 2011 [6]	Prospective observational	Newly diagnosed HER2+ MBC	Trastuzumab versus no trastuzumab	Time to BM progression OS
	Olson et al., 2013 [14]	Meta-analysis	RCTs with adjuvant 1 year trastuzumab reporting BM as first event of metastasis	Trastuzumab versus no trastuzumab	Incidence of BM as first metastatic site Time to BM OS
	Dawood et al., 2008 [15]	Retrospective	HER2 + MBC with BM	Trastuzumab versus no trastuzumab	Time to BM OS after BM
Pertuzumab					
	CLEOPATRA, Swain et al., 2014 [16]	Phase III	HER2+ MBC, first-line	Trastuzumab + docetaxel +/− pertuzumab	Incidence of BM as first metastatic site Time to BM OS
	PATRICIA, Lin et al., 2021 [17]	Phase II	HER2+ MBC, progressing BM after RT	Pertuzumab + high dose trastuzumab	ORR, DOR, CBR
	RePer, Gamucci et al., 2019 [18]	Retrospective	HER2+ MBC, first-line	Trastuzumab + pertuzumab + taxane	ORR, OS
	Esin et al., 2019 [19]	Retrospective	HER2+ MBC, First-line, trastuzumab naïve	Trastuzumab + pertuzumab + taxane	PFS, OS
	PHEREXA, Urruticoechea et al., 2017 [20]	Phase III	HER2+ MBC	Trastuzumab + capecitabine +/− pertuzumab	PFS, OS
T-DM1					
	EMILIA, Krop et al., 2015 [21]	Phase III	HER2+ MBC, previous trastuzumab+taxane	T-DM1 versus Capecitabine + Lapatinib	CNS progression OS
	KAMILLA, Montemurro et al., 2020 [22]	Phase III	HER2+ MBC	T-DM1	ORR, PFS, OS
	Bartsch et al., 2015 [23]	Retrospective	HER2+ MBC with BM	T-DM1	ORR, PFS, OS
	Fabi et al., 2018 [24]	Retrospective	HER2+ MBC with BM	T-DM1	ORR, PFS, OS
	Jacot et al., 2016 [25]	Retrospective	HER2+ MBC with BM	T-DM1	ORR, PFS, OS
Trastuzumab deruxtecan					
	DESTINY Breast01, Modi et al., 2020 [26]	Phase II	HER2+ MBC, previous T-DM1	Trastuzumab deruxtecan	ORR, PFS, OS
	Jerusalem et al., 2020 [27]				
Lapatinib					
	CEREBEL, Pivot et al., 2015 [28]	Phase III	HER2+ MBC	Lapatinib/capecitabine versus trastuzumab/capecitabine	Incidence of BM Time to first relapse in the CNS BM progression
	EGF105084, Lin et al., 2009 [29]	Phase II	HER2+ MBC with BM, previous trastuzumab	Lapatinib monotherapy	ORR, PFS, OS
	Blackwell et al., 2010 [30]	Phase III	HER2+ MBC	Lapatinib +/− trastuzumab	ORR, PFS, OS
	LANDSCAPE, Bachelot et al., 2013 [31]	Phase II	HER2+ MBC with previously untreated BM	Lapatinib + capecitabine	Objective CNS response, PFS
	Metro et al., 2011 [32]	Retrospective	HER2+ MBC	Lapatinib + capecitabine	BM progression OS
	Sutherland et al., 2010 [33]	Prospective observational	HER2+ MBC	Lapatinib + capecitabine	ORR, PFS
Neratinib					
	TBCRC022, Freedman et al., 2019 [34]	Phase II	HER2+ MBC with BM, with/without previous lapatinib	Neratinib + capecitabine	ORR, PFS, OS
	NALA, Saura et al., 2020 [35]	Phase III	HER2+ MBC	Neratinib/capecitabine versus Lapatinib/capecitabine	Intervention for BM Time to intervention for BM
	NEfERT-T, Awada et al., 2016 [36]	Phase III	HER2+ MBC	Neratinib/paclitaxel versus Trastuzumab/paclitaxel	Incidence BM Time to BM Time to BM progression
Tucatinib					
	HER2CLIMB, Lin et al., 2020 [37]	Phase III	HER2+ MBC previous trastuzumab, pertuzumab T-DM1	Trastuzumab/capecitabine +/− tucatinib	ORR, PFS, OS
	Metzger et al., 2020 [38]	Phase I	HER2+ MBC with BM	Trastuzumab + tucatinib	ORR, CBR, PFS
	Borges et al., 2018 [39]	Phase I	HER2+ MBC	T-DM1 + Tucatinib	ORR, PFS
Pyrotinib					
	PHENIX, Jiang et al., 2019 [40]	Phase III	HER2+ MBC	Pyrotinib + capecitabine	PFS
	Lin et al., 2020 [41]	Prospective Observational	HER2+ MBC	Pyrotinib +/− combination	ORR, PFS
**Combination with radiotherapy**					
Trastuzumab	Chargari et al., 2011 [42]	Prospective Observational	HER2+ MBC with BM	WBRT + trastuzumab	ORR, OS Time to BM progression
T-DM1					
	Geraud et al., 2017 [43]	Retrospective	HER2+ MBC with BM	T-DM1 + SRS or WBRT	Local control, ORR
	Stumpf et al., 2019 [44]	Retrospective	HER2+ MBC with BM	T-DM1 + SRS	Toxicity
Lapatinib					
	Lin et al., 2013 [45]	Phase I	HER2+ MBC with BM	Lapatinib + RT	ORR
	Parsai et al., 2019 [46]	Retrospective	HER2+ MBC with BM	Lapatinib + SRS	Local control, OS
	Kim et al., 2019 [47]	Retrospective	HER2+ MBC with BM	Lapatinib + SRS	Local control, ORR, BM-progression rate
	Christodoulou et al., 2017 [48]	Phase II	HER2+ MBC with BM	Lapatinib + WBRT	Objective response

BM: brain metastases, CBR: clinical benefit rate, DOR: duration of response, MBC: metastatic breast cancer, ORR: objective response rate, OS: overall survival, RT: radiotherapy, SRS: stereotactic radiosurgery, T-DM1: ado-trastuzumab-emtansine WBRT: whole brain radiotherapy.

**Table 2:** Ongoing clinical trials for metastatic HER2+ breast cancer with brain metastases.

Clinicaltrials.Gov Identifier	Title	Phase	N	Population	Treatment Arms	Primary Endpoint
**Brain MRI Screening or Monitoring**						
NCT03881605	Routine MRI Screening Versus Symptom-directed Surveillance for BM among Patients with TNBC and HER2-positive MBC: A Single-center Randomized Pilot Study.	N/A	50	TNBC and HER2-positive MBC without symptoms of BM or known asymptomatic BM at study entry.	Arm A: MRI of the brain at baseline, 4 months, 8 months and 12 months. Arm B: Imaging of the brain will take place only if patients develop symptoms that are suggestive of brain metastases.	Feasibility of screening program (secondary endpoints related to BM outcomes)
NCT03617341	Brain Monitoring for High Risk of BM in MBC.	N/A	200	HER2-positive unresectable or MBC with no prior systemic palliative treatment and without symptomatic BM at screening	Arm A: Brain MRI will be taken at the time of initial diagnosis, first- and second-line treatment failure.	Incidence rates of BM in high-risk patients
NCT04030507	Screening MRI of the Brain in Patients with MBC Managed with First/Second Line Chemotherapy or Inflammatory BC Managed with Definitive Intent: A Prospective Study.	N/A	214	BC irrespective of subtype	No intervention arm: No initial MRI screening will be conducted. Experimental arms: 1. Inflammatory breast cancer managed with curative intent: Initial screening MRI and every 6 months for two years. 2. HR+ or HER2+ metastatic BC: Initial MRI and at first systemic progression after study entry. 3. TNBC: Initial MRI and at first systemic progression after study entry.	Neurologic quality of life at 12 months (HR+/HER2- or HER2+ MBC) incidence of symptomatic BM (TNBC). incidence of BM for patients with (inflammatory BC managed with curative intent).
**Combination with Local Treatments (Surgery or Radiotherapy)**						
NCT04582968	A Phase Ib/II Pilot Study of Pyrotinib Plus Capecitabine Combined with Brain Radiotherapy in HER2-positive BC with BM.	Ib/II	47	HER2-positive MBC with measurable BM.	Arm A: Pyrotinib plus capecitabine combined with fractionated stereotactic radiotherapy (FSRT) or WBRT.	Safety and tolerability of pyrotinib Plus capecitabine combined with brain radiotherapy (phase Ib part). Intracranial local tumor control rate (Phase II part).
NCT01494662	A Phase II Trial of HKI-272 (Neratinib), Neratinib and Capecitabine, and T-DM1 for Patients with HER2-Positive BC and BM.	II	168	HER2-positive MBC with BM. Cohort 1: Patients with progressive BM. Cohort 2: Patients who are candidates for craniotomy. Cohort 3a: Lapatinib naïve. Cohort 3b: Prior lapatinib treatment Cohort 4a: Previously untreated BM. Cohort 4b: Progressive BM. Cohort 4c: Progressive BM and prior T-DM1.	Cohort 1: Neratinib. Cohort 2: Neratinib + Surgery. Cohort 3a and 3b: Neratinib + Capecitabine. Cohort 4a, 4b and 4c: Neratinib + T-DM1.	ORR in the CNS by composite response criteria in cohort 1.
**Systemic Treatment (Monotherapy or Combination)**						
NCT03190967	Phase I/II Study of T-DM1 Alone Versus T-DM1 and Metronomic Temozolomide in Secondary Prevention of HER2-Positive BC BM Following Stereotactic Radiosurgery.	I/II	125	HER2-positive BC with BM for which standard curative measures do not exist or are no longer effective.	Arm A: T-DM1 (3.6 mg/kg IV every 21 days) + Temozolomide (30, 40 or 50 mg/m^2^ daily for the phase I).	Maximum Tolerated dose of TMZ when used in combination with T-DM1 (phase I). Median time to progression (phase II).
NCT04512261	TOPAZ: Single Arm, Open Label Phase 1b/2 Study of Tucatinib in Combination with Pembrolizumab Additionally, TrastuZumab in Patients with HER2-Positive BC BM.	Ib/II	33	HER2-positive MBC with untreated or previously treated and progressing CNS disease.	Arm A: Tucatinib + pembrolizumab + trastuzumab.	24-week CNS DCR. Recommended dose of tucatinib in combination with pembrolizumab and trastuzumab.
NCT03765983	Phase II Trial of GDC-0084 in Combination With Trastuzumab for Patients with HER2-Positive BC BM.	II	47	HER2-positive metastatic breast cancer with: Cohort A: Unequivocal evidence of new and/or progressive BM. Cohort B: New and/or progressive BM with clinical indication for resection.	GDC-0084 (45 mg orally once daily) + trastuzumab (8 mg/kg intravenously loading dose; followed by 6 mg/kg IV every 3 weeks thereafter)	ORR in the CNS (RANO-BM criteria).
NCT04752059	Phase II Study of Trastuzumab Deruxtecan (T-DX; DS-8201a) in HER2-positive BC with Newly Diagnosed or Progressing BM.	II	15	HER2-positive MBC with newly diagnosed BM or BM progressing after prior local therapy and measurable disease (RANO-BM criteria).	Trastuzumab deruxtecan 5.4 mg/kg i.v. on day 1 once every three weeks.	Response rate of BM according to RANO-BM criteria.
NCT04760431	Anti-HER2 TKI Versus Pertuzumab in Combination with Trastuzumab and Taxane as First-Line in HER2-positive BC with Active BM: A Phase II, Multicenter, Double-blind, Randomized Clinical Trial.	II	120	Patients of HER2-positive BC with a documented CNS recurrence/progression during or after trastuzumab.	Arm A: Trastuzumab, taxanes and pertuzumab. Arm B: Trastuzumab, taxanes and pyrotinib.	Intracranial ORR.
NCT04158947	A Randomized Study of HER2-positive BC with Active Refractory BM Treated with Afatinib in Combination with T-DM1 Versus T-DM1 Alone.	I/II	130	Patients with HER2-positive BC with documented CNS recurrence/progression during or after anti-HER2 therapy (trastuzumab and/or lapatinib, pyrotinib, tucatinib).	Arm A: T-DM1 and afatinib (Phase I dose escalation starting at 20 mg/day). Arm B: T-DM1.	Safety and tolerability of T-DM1 and afatinib to determine the recommended Phase II dose (RP2D) ORR.
NCT04739761	An Open-Label, Multinational, Multicenter, Phase 3b/4 Study of Trastuzumab Deruxtecan in Patients with or without Baseline BM with Previously Treated Advanced/Metastatic HER2-Positive BC (DESTINY-Breast12).	IIIb/IV	500	HER2-positive MBC Cohort 1: No evidence of BM. Cohort 2: Untreated BM not needing immediate local therapy, or previously treated stable or progressing BM.	Arm A: Trastuzumab deruxtecan 5.4 mg/kg, every 3 weeks.	ORR in participants without BM at baseline (Cohort 1). PFS in participants with BM at baseline (Cohort 2).
NCT04639271	A Single-arm, Open-label Study Of Pyrotinib Combined with Trastuzumab Additionally, Abraxane in Patients with BM from HER2-positive MBC.	II	100	HER2-positive MBC with measurable BM.	Arm A: Pyrotinib Plus Trastuzumab Additionally, Abraxane.	ORR of intracranial lesions PFS of intracranial lesion.

BM: brain metastases, CNS: central nervous system, DCR: disease control rate, MBC: metastatic breast cancer, MRI: magnetic resonance imaging, N/A: non-applicable, ORR: objective response rate, PFS: progression-free survival, RANO-BM: response assessment in neuro-oncology brain metastases. TNBC: triple-negative breast cancer.
